# Evaluation of a community-based intervention for health and economic empowerment of marginalized women in India

**DOI:** 10.1186/s12889-020-09884-y

**Published:** 2020-11-23

**Authors:** Shantanu Sharma, Devika Mehra, Faiyaz Akhtar, Sunil Mehra

**Affiliations:** 1 Department of Clinical Sciences, Lund University, Skåne University Hospital, S-20502 Malmö, Sweden; 2 MAMTA Health Institute for Mother and Child, B-5, Greater Kailash Enclave-II, Delhi, 110048 India

**Keywords:** Health education, Implementation science, Maternal health, Employment, Reproductive health, Health services, Poverty

## Abstract

**Background:**

Empowered women have improved decision-making capacity and can demand equal access to health services. Community-based interventions based on building women’s groups for awareness generation on maternal and child health (MCH) are the best and cost-effective approaches in improving their access to health services. The present study evaluated a community-based intervention aimed at improving marginalized women’s awareness and utilization of MCH services, and access to livelihood and savings using the peer-led approach from two districts of India.

**Methods:**

We used peer educators as mediators of knowledge transfer among women and for creating a supportive environment at the household and community levels. The intervention was implemented in two marginalized districts of Uttar Pradesh, namely Banda and Kaushambi. Two development blocks in each of the two districts were selected randomly, and 24 villages in each of the four blocks were selected based on the high percentage of a marginalized population. The evaluation of the intervention involved a non-experimental, ‘post-test analysis of the project group’ research design, in a mixed-method approach. Data were collected at two points in time, including qualitative interviews at the end line and tracking data of the intervention population (*n* = 37,324) through an online management information system.

**Results:**

Most of the women in Banda (90%) and Kaushambi (85%) attended at least 60% of the education sessions. Around 39% of women in Banda and 35% of women in Kaushambi registered for the livelihood scheme, and 94 and 80% of them had worked under the scheme in these two places, respectively. Women’s awareness about MCH seemed to have increased post-intervention. The money earned after getting work under the livelihood scheme or from daily savings was deposited in the bank account by the women. These savings helped the women investing money at times of need, such as starting their work, in emergencies for the medical treatment of their family members, education of their children, etc.

**Conclusion:**

Peer-led model of intervention can be explored to improve the combined health and economic outcomes of marginalized women.

**Supplementary Information:**

The online version contains supplementary material available at 10.1186/s12889-020-09884-y.

## Background

India achieved groundbreaking success in reducing maternal mortality by 77% from 556 (1990) to 130 per 100,000 live births in 2016 [1, 2]. However, India is still far from reaching the Sustainable Development Goal’s (SDG) target of reducing the Maternal Mortality Ratio (MMR) to less than 70 per 100,000 live births by 2030 [1]. Only three states in India have been able to reduce MMR to less than 70 per 100,000 live births so far [3]. In the move to accelerate the pace of achievement of the SDG, NITI Aayog (the think tank of the Government of India) has spearheaded the health index initiative with the Ministry of Health and Family Welfare. Nearly half of the Indian states or union territories had an index score of 50 or less than 50 out of 100 in the health index initiative [4]. This reflects poor health outcomes and health system performance and service delivery indicators across half of the states or union territories in India [4].

The Government of India adopted multiple strategies to improve maternal health and reduce MMR in the Millennium Development Goals’ era. These strategies included improving access to quality maternal health services, introducing conditional cash transfer schemes, such as maternity benefit scheme and cashless delivery scheme, mitigating social determinants of maternal health, and promoting public-private partnerships [1]. The evidence state that increased access by women to quality maternal and perinatal health services is essential for improving maternal and perinatal outcomes, including reduced maternal deaths [5].

It is well known that social and structural determinants of maternal health influence the access to and use of maternal and reproductive health care services [6, 7]. Studies indicate that social and economic disparities critically affect the utilization of health services amongst other determinants of maternal health [8, 9]. Socially disadvantaged populations are often challenged by long distances to health facilities, poverty, ignorance, and poor health literacy [10, 11]. Other studies have shown that woman’s educational attainment, age at marriage, access to livelihood, access to savings, and decision-making power influence the utilization of health services [12–15].

Community-based interventions that disseminate education and promote awareness related to maternal and child health care, and based on building women’s groups (peers) are best for improving access to health services [16]. Peers in the communities have an important role to play in transforming the health practices of women. Multiple studies have proven the effectiveness of peers in community-based interventions to educate women and improve the health status of the mothers and children [17–19]. Community engagement is central to community-based interventions and a cost-effective tool for sustained behavior change [16, 20]. Community engagement has been a widely used health promotion strategy, involving communities in decision-making, planning, and delivery of services [21]. It has been widely used for marginalized populations as it engages with the community at all levels, enables knowledge transfer exchanges, and addresses power imbalances [22].

While the effect of peer-based interventions in improving awareness and utilization of maternal and child health services has been well described, most of these interventions are limited to health education approaches. There is limited evidence for the effect of peer-based interventions in jointly improving awareness and utilization of maternal and child health services, and access to livelihood and savings using education and livelihood promotion approaches. Therefore, we implemented a peer-led community-based intervention in a holistic approach for changing not only the knowledge and practices of women but also reducing the socio-economic inequities by making the services more inclusive and improving the livelihood opportunities in the villages. In the three-year intervention of behavior change, we engaged with relevant stakeholders influencing the decision-making and utilization of maternal health services, particularly husbands, mothers-in-law, community health workers, and community gatekeepers, besides women themselves.

We conducted the present study to identify perceived changes in the awareness and utilization of maternal and child health services and access to livelihood and savings after a three-year intervention. Furthermore, we wanted to assess the change in the perceptions and practices of the relevant stakeholders influencing the uptake of maternal and child health services and livelihood by women. This paper shows the mixed-method evaluation of this community-based intervention from two districts in India.

### Description of the intervention

#### Intervention settings

**Fig. 1:**
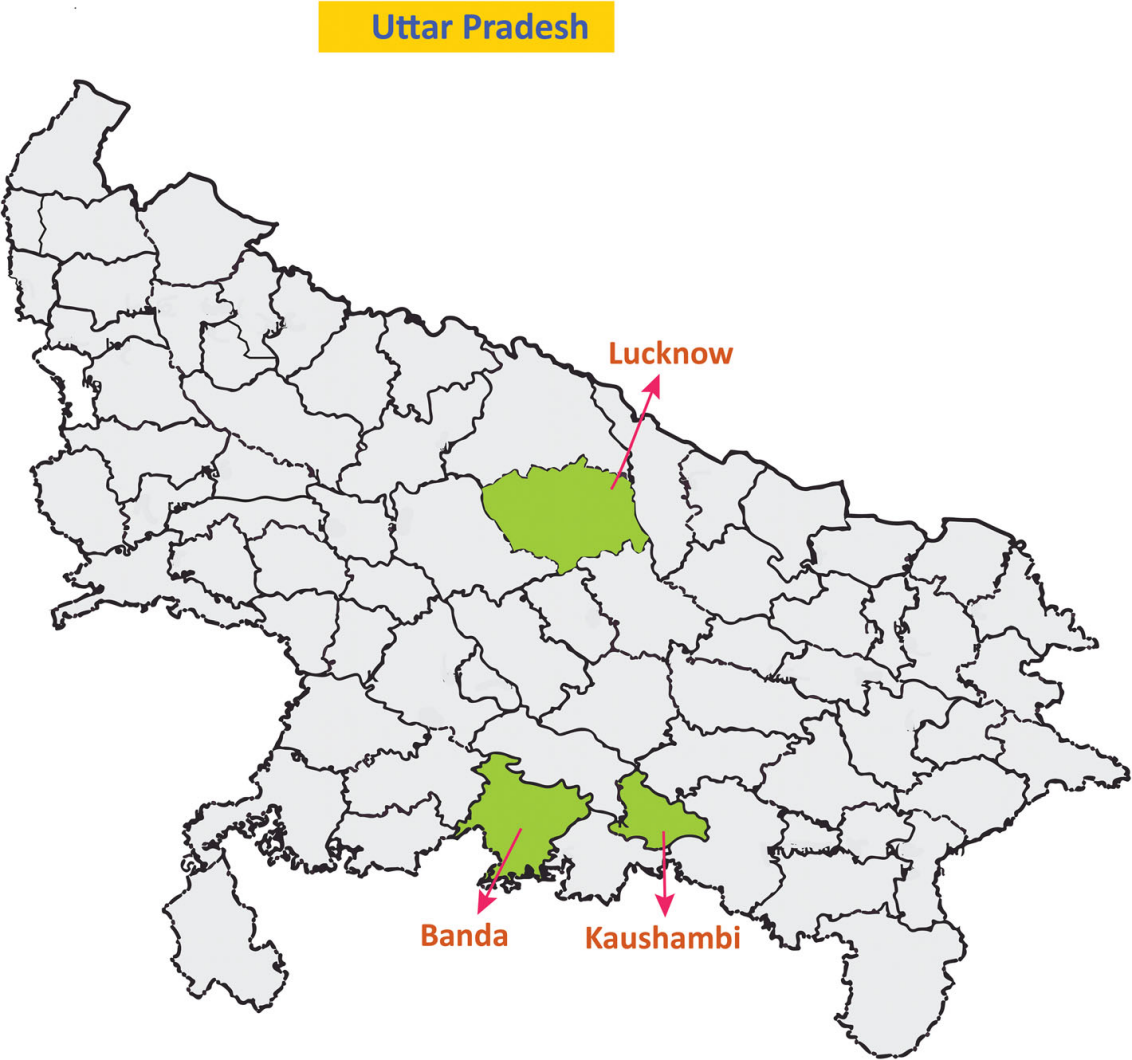
Location of the two districts of the intervention in the map of Uttar Pradesh (the state in the central zone of India). Banda is situated to the south and Kaushambi to the south-east of the capital of Uttar Pradesh (Lucknow). The map depicted above was developed by us

The project was implemented in two marginalized districts of Uttar Pradesh, namely Banda and Kaushambi. Uttar Pradesh, an empowered action group state, lags behind most of the states in the country in terms of the major indicators of health, social, and economic development [23]. The two districts (Banda and Kaushambi) were chosen randomly from the list of the districts in Uttar Pradesh falling under the category of low or very low human development index [23]. Banda is situated to the south of the capital of Uttar Pradesh (Lucknow) and has a population of 1.8 million, 66.6% literacy rate, and a sex ratio of 863 [24]. Situated south-east to the capital of Uttar Pradesh, the district Kaushambi has a population of 1.6 million, 61% literacy rate and a sex ratio of 908, which is lower than the state and national averages [24]. The location of the two districts in the map of Uttar Pradesh is shown in Fig. 1. Two development blocks in each of the two districts were selected randomly for the intervention. All the villages in the selected blocks were ranked based on a higher percentage of the marginalized population (defined as population belonging to scheduled caste, scheduled tribe, other marginalized classes, or below the poverty line). The top 24 villages in the list from all the four blocks were chosen for the intervention. In total, the intervention was conducted in 96 villages from four blocks.

#### Objectives of the intervention

The specific objectives of the intervention were:

- to identify, map, and organize groups of women (15–35 years) from marginalized sections of society;
- to orient community health workers, midwives, and medical officers on segmentation and inclusive approach [25] for delivering focused care to marginalized populations on the issues of maternal and child health, gender, life-skills, and savings;
- to deliver health education sessions to women from marginalized sections using a peer-led approach with the support of community health workers and midwives; and
- to create an enabling environment for increasing women’s access to health and livelihood by sensitizing community leaders, employment office staff, husbands, and mothers-in-law. Access to livelihood was assessed by linkage with a government-sponsored wage employment scheme, Mahatma Gandhi National Rural Employment Guarantee Act (MGNREGA scheme), which aims at providing livelihood security to the rural poor [26]. MGNREGA scheme supports livelihoods by guaranteeing cash payments for work. Under the scheme, every household has a legal right to access 100 days of work per year to generate public assets, such as water harvesting structures, irrigation facilities, and other livelihoods infrastructure intended to benefit communities [27]. Access to savings was assessed as having an account in a bank or in a post-office in our study.

**Fig. 2:**
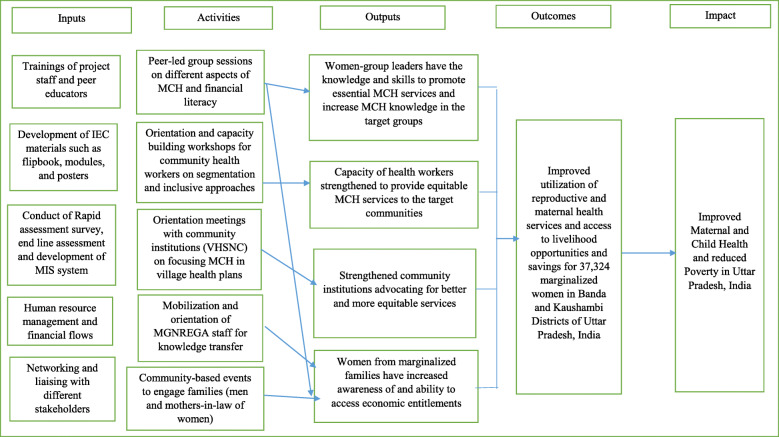
Theory of change for the community-based intervention across two districts of Uttar Pradesh, India. The theory of change model illustrates how the desired outcome of improved utilization of reproductive and maternal health services and access to livelihood opportunities and savings for 37,324 marginalized women in Banda and Kaushambi Districts of Uttar Pradesh, India was achieved through precise link of inputs, activities, and outputs. Abbreviations: IEC: Information, education, and communication, MIS: Management information system; MCH: Maternal and child health; MGNREGA: Mahatma Gandhi National Rural Employment Guarantee Act; MCH: Maternal and child health; VHSNC: Village health, sanitation, and nutrition committee

The theory of change for the intervention based on the community engagement model is shown in Fig. 2. The proposed theory of change in our intervention was to improve awareness and utilization of maternal and child health services, and access to livelihood (MGNREGA) and savings by the marginalized women. In the community engagement approach, we used peer educators as mediators of knowledge transfer and created a supportive environment at the household and community level. In addition, to make this entire change sustainable, the strengthening of Village Health, Sanitation, and Nutrition Committees (VHSNC) was done. VHSNC are entrusted as a key mechanism for community health governance to ensure community participation in monitoring and quality delivery of health and nutrition services. Strengthening VHSNC is a step towards ensuring community engagement and sustainability in the change process of the community [28].

#### Intervention activities

The sequential flow of the intervention activities is shown in Table 1. The project aimed to reach up to a total of 37,324 marginalized women from the two districts. The groups of identified women were formed with each group containing 23–27 women. From within the group, one woman was selected as a peer educator, educated up to high school (minimum qualification) and had leadership skills (assessed by outreach worker). There were 1500 peer educators across both districts. Outreach workers trained all the peer educators on a four-day module covering ten topics. These ten topics were related to maternal and child health and financial literacy (saving money, managing household expenditure, and opening an account). Similarly, peer educators conducted sessions with women in the community on these ten topics. Peer educators paid home visits to women at the time of recruitment and in case of drop-outs during the intervention. Since peer educators belonged to the same community, they had access to approach these women anytime (the exact number of home visits not recorded). Based on the intervention model, the various implementation actors were assigned their roles (Table 2).

**Table 1:** Sequential flow of the implementation activities

Phases of Implementation	Implementation activities
Pre-intervention	1. Rapid assessment survey at the baseline 2. Mobilization of marginalized women from the communities and formation of groups by outreach workers 3. Identification of peer educators from within the groups and training of peer educators by outreach workers
Intervention	1. Education sessions on maternal and childcare and financial literacy by peer educators 2. Community-based events and meetings to engage community leaders, husbands, and mothers-in-law of young married women 3. Active participation in the village health sanitation and nutrition committees’ meetings 4. Capacity building of community health workers and midwives on segmentation and inclusive approach for delivering services related to sexual and reproductive health 5. Linking marginalized women with MGNREGA schemes, and running a campaign of ‘*kaam mango abhiyan*’ (Work demand campaign).
Post-intervention	End line assessment

*Abbreviations*: *MGNREGA* Mahatma Gandhi National Rural Employment Guarantee Act

**Table 2:** Implementation actors and their roles

Implementation actors	Roles
State Manager and District coordinators	Effective liaising with government, and other relevant stakeholders, monitoring the activities, reviewing the progress of the intervention and managing the challenges and risks at the local level
Outreach workers	Conducting the training and providing mentoring support to the peer educators, handholding support and liaising with community health workers, VHSNC members, and monitoring the activities of peer educators
Peer educators	Conducting education sessions for peers in the communities, handling problems, and providing solutions to their problems

*Abbreviations*: *VHSNC* Village health sanitation and nutrition committee

Various information, education, and communication tools were developed and used in the intervention: a flipbook with illustrations and key messages, a training module of ten chapters, and ten posters on maternal and child health [29]. All the education tools were in the local language (Hindi). The cover page and table of contents’ page of the flipbook and module are shown as supplementary Figures 1 and 2 (Supplementary file 1), respectively. All the education tools were tested for relevance, face, content, and construct validity; the mention of those is beyond the scope of this paper. The sessions in flipbooks or chapters in the module included information on pre-pregnancy, antenatal, postnatal, and newborn care, child immunization, nutrition and growth, family planning, and financial literacy. The modules were meant for training of peer educators by outreach workers, and flipbooks were intended for use by the peer educators in the field while taking group sessions. The posters were intended to be used during community-based events. MAMTA’s technical committee reviewed the educational tools.

The duration of each session by a peer educator in the field was 2 h. The sessions began with a 20-minute-discussion on the pictures shown in the flipbook followed by 60–80 min of discussion on the key messages related to the topic, and in the end, participants could ask queries. A question box was also kept in each session for the participants hesitant to discuss their issues in front of their peers. Women were advised to save money monthly in a piggy bank so that the saved money could be used for meeting the expenditures during delivery and the postnatal period.

The outreach workers facilitated the linkages of these women with the MGNREGA scheme. The community health workers, midwives, and MGNREGA staff were mobilized and oriented to sustain the process of knowledge transfer among marginalized communities as well as increased mobilization towards a better uptake of social-welfare schemes. The medical officers in the intervention areas were sensitized on an inclusive approach to delivering appropriate and quality maternal and child health services. The members of VHSNC were oriented and engaged for better coordination between community and health systems in addressing the needs of marginalized populations. Further, VHSNC were strengthened to prioritize the issues related to maternal and child health in their village health plans.

#### Rapid assessment at baseline

We conducted a mixed-method rapid assessment at the baseline to explore maternal and child health-related awareness and service utilization (antenatal, intra-natal, and postnatal) among marginalized women. The data were collected at baseline from January to March 2013. The data collection was done on a sample of the population from the intervention sites by outreach workers of the project.

The pre-validated questionnaire used for the quantitative survey among young women (15–35 years) included questions on their socio-demographic characteristics, awareness on maternal and child health care (antenatal care, anemia, maternity benefit scheme, postnatal care, family planning methods, home-based care for diarrhea in new born, symptoms of pneumonia, child immunization, and safe abortion), awareness on national health insurance and MGNREGA schemes. Further, information on the possession of bank or post-office account and work participation by the women under the major income-generating scheme of the government of India (MGNREGA) was collected. In addition, we asked about the utilization of maternal and child health services (antenatal care, institutional delivery, postnatal care, early breastfeeding, child immunization, abortion, and family planning methods).

Qualitative interviews were conducted to gain our understanding of the needs and perceptions of young married women (15–35 years) and the service providers on maternal and child health and family planning. In each district, one focus group discussion (FGD) was conducted, each with pregnant women, lactating women, and community health workers, and one in-depth interview (IDI) was performed with a medical officer. In addition, two IDI were done with the staff of the MGNREGA scheme to understand their perceptions about women’s participation in MGNREGA (one in each district).

We employed multi-stage random and purposive sampling techniques to recruit participants for the quantitative survey and qualitative interviews, respectively, in the rapid assessment survey. Based on the data from this assessment of 3 months, we developed the intervention protocol and set a list of activities that were potentially effective and required to achieve the objectives. The mean number of participants in the focus groups was 10–15.

#### Findings of the rapid assessment study

Of 476 young married women, 240 from Banda and 236 from Kaushambi, who were pregnant or had a child less than 3 years of age, responded to the quantitative questionnaire. The mean age of the women was 27 years. Approximately 66 and 81% of the women were illiterate (could not read and write) in Banda and Kaushambi, respectively (Supplementary Table 1; Supplementary file 2). Overall, the socio-economic and demographic characteristics of the women were poor. Less than 50% of the women were aware of anemia, maternity benefit schemes, postnatal care, safe places for abortion, and the MGNREGA scheme (Supplementary Table 2; Supplementary file 2). More than three-fourths of the women did not receive three or more antenatal check-ups in both districts. The institutional delivery rate was 78 and 64% in Banda and Kaushambi, respectively. Only 23% of women in Banda and 13% in Kaushambi were using any family planning method at the time of the survey. Around 40–46% of women had bank accounts, and less than one-third of the women were registered for the MGNREGA scheme. Only 13% of women in both districts had ever worked under the MGNREGA scheme.

Although both districts had poor socio-economic indicators, Kaushambi was found to have a higher burden of poverty, illiteracy, and unemployment than Banda. The qualitative analysis was broadly divided into three key themes, namely, perceived awareness and utilization of maternal and child health services, perceived support from family (husbands or mothers-in-law), and utilization of the MGNREGA scheme by women (Table 3). Some of the key issues highlighted during qualitative analysis were perceived poor knowledge about the importance of antenatal check-ups, family planning methods, newborn and childcare, perceived dissatisfaction with the government health services, and limited access to the MGNREGA scheme among women.

**Table 3:** Qualitative data analysis from the rapid assessment at baseline

Themes	Responses obtained from the interviewees
Perceived awareness and utilization of MCH services by women	**a. From the discussions with women** Women appeared to have little information on newborn and child care practices, including breastfeeding, and newborn hygiene. Their utilization of antenatal services was perceived poor. Most of the women seemed to have been dependent on mothers-in-law for any health-related problem of their children. *We visit doctors only in case of acute illness and not routinely for an antenatal check-up during pregnancy*. *We are poor and illiterate women who do not know about medical check-ups during pregnancy. I have not been told or asked by anyone to go to the doctor for a check-up*. (Women during FGD) *There is an exploitation of poor people at the PHCs. The medicines prescribed need to be purchased from private medical stores. If there are good medicines at PHC, we will surely buy from there for our treatment*. (Women during FGD) *My child suffered from pneumonia, and I took him to the government hospital, but there was no relief. Then, I took my child to a private hospital where my child fully recovered.* (Women during FGD) A few women seemed to know about family planning methods. However, the uptake of contraceptives was perceived poor because women were hesitant to talk about them, they appeared to have limited knowledge about them, a lot of misconceptions about their side effects prevail in the society, women seemed to lack negotiation skills, non-cooperating attitude of husbands, and unavailability of contraceptives at health facilities. **b. From the interviews with healthcare providers** Medical officers revealed that the institutional delivery rate has improved in their areas, which might be due to the launch of the maternity benefit scheme (*Janani Suraksha Yojna*; JSY). Community health workers informed that women preferred to deliver in a district-level hospital instead of a PHC because of a lack of adequate facilities and pediatricians for the care of the newborn in PHCs. *JSY has a big role in promoting institutional delivery at government service centers*. (A medical officer during IDI)
Perceived support from family (husbands or mothers-in-law) for accessing MCH services	Women in some of the FGD reported a lack of support from husbands for institutional delivery or to work outside their homes. *Husbands denied permission for delivery at PHC and did not want money given under the JSY scheme.* (Women during FGD; JSY is maternity benefit scheme) *Men in the community wanted delivery to happen at home. They went to doctors only if their wives developed some problems after delivery*. (Women during FGD) *Our society is a male-dominated society. In some cases, it’s a male’s ego, which does not allow men to let their wives work outside their home and earn some money.* (Women during FGD)
Perceived utilization of the MGNREGA scheme by women	A fewer number of women went to work under MGNREGA. Many women who started working under MGNREGA left it once they became pregnant. The MGNREGA staff mentioned that various services were provided to pregnant women at the worksites such as drinking water and medicines, a shed to feed their children. However, most of the women did not continue for a very long time. *Yes, the women may feel difficult to work at MGNREGA sites because of their pregnancy or because they had delivered a baby. Repeated childbirths deprive them of good health and stamina, and the mothers are not able to produce output at the work sites*. (A MGNREGA staff)

*Abbreviations*: *FGD* Focus group discussion, *IDI* In-depth interviews, *JSY* Janani Suraksha Yojna, *MGNREGA* Mahatma Gandhi National Rural Employment Guarantee Act, *MCH* Maternal and child health, *PHC* Primary health centers

## Methods

### Evaluation design

The evaluation of the intervention involved a non-experimental ‘post-test analysis of the project group’ research design in community settings, in a mixed-method approach. No control group was present. The intervention was delivered over 30-months from the start of April 2013 until the end of September 2015. The post-intervention qualitative data were collected and analyzed at the end of the intervention in October and November 2015.

### Data collection

The end line data collection was done on a sample of the population from the intervention sites. In addition, we tracked the intervention population (*n* = 37,324) on certain quantitative indicators, and their data were captured in the routine management information system. The evaluation was conducted on all the relevant stakeholders engaged in the intervention, who provided consent and were available for the interviews. The stakeholders included young married women (15–35 years), peer educators, husbands and mothers-in-law of women, community health workers, midwives, medical officers, VHSNC members, and MGNREGA staff. Outreach workers of the project collected the routine monitoring data. However, the end line qualitative research was done by a team from the Department of Community Medicine, Institute of Medical Sciences, Banaras Hindu University, Varanasi.

#### Management information system data

An online management information system was established. Young married women (*n* = 37,324) were tracked for selected indicators, including socio-demographic variables (age at enrollment in the study, age at marriage, education, religion, social class, and possession of below poverty line card) and attendance in group education sessions. Five outcomes obtained through the management information system were compared with the baseline data. These included a) status of the last delivery (institutional), b) if women had received postnatal care within 48 h of delivery, c) if women had an account in a bank or in a post-office, d) if women registered to work under MGNREGA scheme, and e) if women utilized the MGNREGA scheme. All the data collected on papers by the outreach workers during the intervention were entered regularly into the management information system. The intervention progress was monitored at three-time intervals (after the first 15 months, during the middle 12 months, and in the last 8 months) using four indicators, including the percentage of women identified, women groups formed, peer educators trained, and women accessed by community health workers or midwives. The progress was monitored based on the data entered into the information system online on a routine basis.

#### End line assessment

We conducted qualitative research at the end line to observe perceived changes in the outcomes of the project. Purposive sampling was done to recruit participants. FGD and IDI were the primary data sources. A total of 12 FGD were conducted with women (*n* = 4), peer educators (*n* = 2), mothers-in-law (*n* = 2), outreach workers (*n* = 2), and community health workers (*n* = 2). Furthermore, 22 IDI were conducted with all the other stakeholders, including husbands of women (*n* = 4), midwives (*n* = 2), medical officers (*n* = 2), district coordinator of the project (*n* = 2), and VHSNC members (*n* = 4), community health workers (*n* = 4) and peer educators (*n* = 4). The samples were distributed equally across both districts. We used semi-structured interview guides during the interviews (see the interview guides; Supplementary file 3).

### Ethical considerations

Ethical approval for the study was obtained from the MAMTA Institutional Ethical Review Board. Verbal informed consent was obtained from all the participants. All the participants were ensured of the confidentiality of the information shared with the project staff and research teams.

### Data management and analysis

#### Quantitative analysis

Data were expressed as frequencies and percentages for categorical variables and mean (Standard Deviation) or median for continuous variables. IBM SPPS statistics for windows version 24.0 (IBM Corp., Armonk, N.Y., USA) was used to do the analysis.

#### Qualitative analysis

All the interviews at end line assessments were recorded, transcribed, and translated into English. A thematic analysis was carried out to identify the main patterns related to the responses, and coding was done. All transcripts were assessed by two researchers and refined into codes inductively. The themes generated were reviewed, refined, and discussed among researchers for consensus validation. Discrepancies among the researchers in interpretation were resolved through discussion, which helped further develop the analysis. The analysis emerged into the following thematic areas:

- Perceived changes in the awareness and utilization of maternal and child health services by women,
- Perceived changes in economic independence in households and livelihood opportunities for women,
- Perceived changes in gender equity norms in the societies,
- Perceived changes in the functioning of VHSNCs, and
- Peer educators’ and health care provider’s experiences and perceptions of the changes in the community

## Results

The results have been divided into two sections: the analysis of the data obtained from the management information system and end line qualitative assessment.

### Management information system data

The socio-demographic characteristics of the intervention population tracked through the management information system is shown in Table 4. Most women in Banda (90%) and Kaushambi (85%) attended at least 60% of the education sessions. Around 39% of women in Banda and 35% in Kaushambi were registered under the MGNREGA scheme, and 94 and 80% of them had also worked in it, respectively (Table 5).

**Table 4:** District-wise distribution of the socio-demographic characteristics of women (*n* = 37,324) obtained through the management information system

Socio-demographic variables	Banda (*n* = 18,871) N (%)	Kaushambi (*n* = 18,453) N (%)
Mean (SD) age of women (years) at the time of enrollment^a^	28 (8.4)	28 (9.2)
Mean (SD) age of women at marriage (years)^b^	17.3 (1.4)	14 (7.3)
Mean (SD) years of schooling (years)^c^	4 (3.0)	10 (1.5)
Religion		
Hindu	17,845 (94.6)	17,010 (92.2)
Muslim	1018 (5.4)	1432 (7.8)
Christian	8 (< 0.1)	2 (< 0.1)
Missing cases	0	9 (< 0.1)
Social class		
Scheduled castes	7084 (37.5)	16,463 (89.5)
Scheduled tribes	1139 (6.0)	94 (0.5)
Other marginalized castes	10,195 (54.0)	1446 (7.9)
Non-marginalized class	408 (2.2)	133 (0.7)
Missing cases	45 (< 0.1)	317 (1.7)
Possession of below poverty line card		
Yes	12,341 (65.4)	7766 (42.3)
No	6530 (34.6)	10,608 (57.7)
Missing cases	0	79 (< 0.1)

*Abbreviations*: *SD* Standard Deviation

^a^The denominator for Banda was 14,984 and Kaushambi was 16,655

^b^The denominator for Banda was 18,869 and Kaushambi was 18,447. The data were skewed. The median years of schooling for Banda were 0

^c^The denominator for Banda was 18,861 and Kaushambi was 18,453

**Table 5:** Frequency and percentage distribution of the outcome indicators comparing data from baseline and Management information system

Variables	Baseline		MIS Data	
	Banda (*n* = 240) N(%)	Kaushambi (*n* = 236) N(%)	Banda (*n* = 18,871) N(%)	Kaushambi (*n* = 18,453) N(%)
Women who had an account in her name in bank or post-office	93 (38.8)	108 (45.8)	10,316 (54.7)	8238 (44.6)
Women registered to work in MGNERGA	73 (30.4)	67 (28.4)	7353 (39.0)	6591 (35.7)
Of those registered to work, women who utilized MGNREGA scheme^a^	31 (42.4)	32 (47.7)	6922 (94.1)	5234 (79.4)
Women who delivered in an institution^b^	189 (79.1)	162 (68.6)	794 (97.7)	95 (87.2)
Women who received postnatal care within 48 h of delivery^c^	31 (12.9)	26 (11)	749 (78.0)	85 (78.0)

*Abbreviations*: *MGNREGA* Mahatma Gandhi National Rural Employment Guarantee Act, *MIS* Management Information System

^a^Total sample size for MIS data for Banda (*n* = 7353) and Kaushambi (*n* = 6591)

^b^Total Sample size for MIS data was: Banda (*n* = 847) and Kaushambi (*n* = 109)

^c^Total sample size for MIS data was: Banda (*n* = 1629) and Kaushambi (*n* = 1372)

Nearly 24% of women had opened accounts as well as were registered in the MGNREGA scheme (Table 6). The progress tracked through the management information system at three-time intervals (after the first 15 months, during the middle 12 months, and in the last 8 months) has been shown in supplementary Figure 3 (Supplementary file 1).

**Table 6:** Frequency and percentage distribution of women who had accessed/not accessed MGNREGA and/or had a bank account and/or received education sessions based on MIS data

Variables	Total (*n* = 37,324) N(%)
Women who registered in MGNREGA and had a savings bank account	8932 (23.9)
Women who registered in MGNREGA but did not have a savings bank account	5012 (13.4)
Women who had a savings bank account but did not register in MGNREGA	9622 (25.8)
Women who did not register in MGNREGA and did not have a savings bank account, but have received group education sessions and taken any other health benefits	13,758 (36.8)

*Abbreviations*: *MGNREGA* Mahatma Gandhi National Rural Employment Guarantee Act, *MIS* Management Information System

### End line assessment

In the FGDs with various groups, the number of participants ranged between 8 and 13. There were 8, 10, 12, and 13 participants in four FGDs with women, 11 and 12 participants in the two FGDs with mothers-in-law, 9 and 10 participants in FGDs with community health workers, 7 and 8 participants in FGDs with outreach workers, and 9 and 12 in FGDs with peer educators. The mean duration of the focus group discussions was 75 min (range: 60–90 min). All the focus groups were physically conducted at a suitable place in villages. The complete analysis of the qualitative data collected at the end line is broadly divided into six themes.

1. Perceived changes in the awareness and utilization of maternal and child health services by women

Women’s awareness of maternal and child health seemed to have increased post-intervention. Most of the women accessing facilities for antenatal care appeared to know about maternity benefit schemes. It appeared from the interviews that they knew about early newborn care practices, the importance of early and exclusive breastfeeding, and a minimum gap of 3 years between two pregnancies. In addition, the awareness regarding consumption of iron-folic acid (IFA) seemed to have increased post-intervention, but it did not appear from the interviews that the consumption had also increased.

Midwives, community health workers, and VHSNC members reported in the interviews that the awareness of the women about health care services had increased. The interviews seemed to indicate that mothers-in-law understood their responsibilities of supporting and caring for their daughters-in-law during pregnancy. It appeared that they knew about the emergency helpline number for the ambulance, the importance of childhood immunization, and family planning.

*Now, all of us go to the hospital for delivery. ASHA bahu comes and takes us to the hospital and cares for us. She calls the vehicle (ambulance), and then we go to the hospital.* (A woman during FGD; community health worker is called ASHA in the community)

*After the intervention, I could perceive the change in the nutritional practices of women. Women have become more caring for their children and maintain hygiene. (A VHSNC member during IDI)*

Peer educators had counselled women on the importance of breastfeeding and maternal nutrition during pregnancy and lactation. Peer educators had accompanied these women to the facilities for further support on many issues such as family planning methods, adequate latching during breastfeeding, and resolving misconceptions regarding immunization. Peer educators perceived that the utilization of most of these services had increased post-intervention. One noticeable change that seemed to have been highlighted in the interviews was that the demand for health services from the communities had increased. However, it appeared from the interviews that the uptake of contraceptives by the women did not change significantly.

It seemed that outreach workers and community health workers had faced challenges in mobilizing marginalized women to avail health services. Some of the major challenges that prompted low utilization of health services among marginalized women before the intervention included illiteracy, poverty, ignorance, lack of women empowerment, traditional beliefs or misconceptions, and long distances to the health facilities. The interviews seemed to indicate that outreach workers and community health workers succeeded in mobilizing such women for the intervention through repeated meetings and counselling of their family members. The gap in the service utilization rates between women from non-marginalized and marginalized families seemed to have decreased after the intervention, as revealed in interviews with community health workers, midwives, and medical officers.

(2) Perceived improvement in economic independence in the households and livelihood opportunities for women

Women who worked under the scheme appeared to be feeling financially strong and independent. The results seemed to indicate that the money earned after getting work under MGNREGA or saved from daily savings was deposited in the bank account by the women. These savings appeared to have helped women investing money at times of need, such as starting their work, in emergencies for the medical treatment of their family members, education of their children, etc. It seemed from the interviews that being an earning member of the family; such women could voice their demands and make decisions for family and self.

*After being associated with MAMTA, I was encouraged by family members and neighbors to go outside and work. I worked at a place where my neighbors were also working; both got the opportunity to work under MGNREGA.* (A woman during FGD)

Women were engaged in jobs other than MGNREGA, such as small cottage industries like poultry, goat rearing, and grocery stores. Some women’s groups opened stitching centers to give training to the girls on stitching and embroidery. Such women’s groups had linked women to other schemes for women’s development. The money earned from the new job was spent on starting a small new business.

*After being associated with MAMTA, I started a stitching center. I used to train other girls. Recently, I have another embroidery and stitching center.* (A woman during FGD)

It was perceived from the interviews that outreach workers and VHSNC heads endorsed this improvement in social mobility and the financial status of women in the communities. An increased number of women and people seemed to have been linked to the MGNREGA scheme, and they received work for a minimum of 50–100 days. VHSNC members informed women that they could work under MGNREGA and earn 156 INR (3USD) for working 8 h a day. Women were also informed that they could borrow money at a low-interest rate from the group’s bank account to start a new venture. Community health workers appeared to have helped women open accounts in banks to transfer the money received under the scheme and had encouraged these women for daily savings and kept the money in piggy banks.

Peer educators seemed to have extended their support to women during the ‘Demand for Work (*Kaam Mango Abhiyan*) campaign,’ and helped them to open bank accounts. In the ‘Demand for Work’ campaign, group meetings were called by the MGNREGA staff to give information about the scheme.

### Other perceived changes

(3) Perceived change in gender equity norms in the communities

Gender disparities regarding access to education, adequate nutrition, and mobility seemed to prevail in the communities. Outreach workers had educated communities about the importance of girls’ education and a nutritious diet for women and girls through magic shows, plays, and community-based events such as ‘*Saas Bahu Sameelan’* (meetings with mothers-in-law and daughters-in-law on a common platform).

*People did not like a girl child in our village. My husband asked my mother-in-law not to give me food because I delivered a baby girl in my previous pregnancy. However, the project has changed the mindset of people. MAMTA staff educated us about the benefits of a girl child and to continue their education and let them earn name and fame. People now don’t consider the difference between a male and a female child*. (A woman during FGD)

Community health workers perceived a positive change in the attitude of men towards their wives. Ration cards (subsidy cards) were issued in women’s names. All the subsidies and incentives from maternity benefit schemes or MGNREGA were transferred into women’s accounts. The interviews indicated that community health workers did not find any reported case of female foeticide in the last 6 months from the date of the interview. The VHSNC head recalled a play organized by outreach workers based on female foeticide and preventing the killing of a girl child. Husbands received education on gender equality and the need to educate girls through magic shows.

*I do agree that if girls are educated, they will know their rights. And now, emphasis should be laid on educating more and more girls. When girls are educated, they will get to know their rights. Hence, education is very important. I do agree that there should be no discrimination between girls and boys. The MAMTA staff had explained to me that one should not go for the gender identity test of the fetus. Craving to have a boy, I have seen people giving birth to six girls.* (A husband during IDI)

*Because of the project, I have observed a change in the attitude of my husband. Earlier I was not allowed to go outside anywhere except for defecation. However, after my husband attended meetings taken by the MAMTA staff, I have noticed a change in his behavior. I could go and move around in my village, and my husband did not mind. I could talk to people easily, chit-chat with other ladies. So, I have got this kind of freedom*. (A woman during FGD).

(4) Perceived changes in the functioning of Village Health Sanitation Nutrition Committees (VHSNC)

The VHSNC members seemed to have not been clear about their role in VHSNC before and appeared to have become aware of the processes after the intervention. They seemed to have started participating actively in the meetings to facilitate the processes of government schemes. The meetings appeared to have been conducted monthly, and it was decided to judiciously use the fund (10,000 INR) to provide health-related facilities in the village. The fund account seemed to have been operated and maintained jointly by the VHSNC head and midwife. In the past, lack of effective communication or coordination between VHSNC members and the midwife appeared to have resulted in the cancellation of the meetings, an issue which was resolved after the intervention. Issues most commonly raised during such meetings included hygiene in the villages, facilitating access to the MGNREGA scheme by the women and people from marginalized communities, and immunization of under-five-year-old children.

*There is a committee controlled by the midwife and VHSNC members to give more facilities to mothers. We channelized funds for cleanliness and support to the poor families who could not bear the expenses related to the delivery of a woman. In some cases, money was collected through group charity. MAMTA staff always encouraged women for better health services.* (A VHSNC head during IDI)

(5) Peer educator’s experiences from the project and perceptions towards changes in the community

Peer educators did home visits, built rapport with family members, and organized sessions with women in the communities. They demanded more training on issues such as the national family health insurance scheme, and refresher training on the other issues. Sessions elaborating on women’s reproductive health and rights, financial literacy, and postnatal care were difficult to discuss by peer educators. The interviews indicated that peer educators found the training, conducted by the outreach workers, useful and engaging because different infotainment materials were used, such as posters, videos, role-plays, and songs for discussions on the topics.

From the interviews, it appeared that as a result of prevailing notions and misconceptions, peer educators found it difficult to obtain consent from the families for engaging their women in different activities. However, the communication tools such as magic shows and demonstrations, interactive meetings with husbands or mothers-in-law, collective decision-making, and feedback mechanisms were some of the key strategies that seemed to have helped mobilize communities for greater engagement in activities and bringing change in women’s health practices. An increase in perceived self-respect, confidence, and improved access to government health schemes was noticed among peers. Peer educators appeared to have helped VHSNC monitor services in the communities for improvement in their quality of work.

*We had observed service delivery points like Anganwadi centers and sub-centers for health services by midwives to assess the availability of different materials.* (Peer educators from FGD; Child development centers are called *Anganwadi* centers in villages)

(6) Health service providers’ experiences from the project and perception about changes in communities

Community health workers and midwives in their interviews affirmed about the high quality of the training and workshops.

*Very good training was given by project staff using different techniques like magic shows, dhol (drums), and TV shows*. (A community health worker during IDI)

*The training was good. Information about all the government schemes was provided. They answered our queries and explained things through posters or TV (shows)*. (A midwife during IDI).

Community health workers perceived increased access to maternal health services by women. However, the consumption of IFA tablets was perceived to be poor. Midwives opinioned that despite hard-core interventions (sessions and meetings), the uptake of family planning and postnatal care services by the women appeared to be poor in the communities. There was a scope of improvement in the intervention, including advocating for the availability of adequate resources in the health centers such as a stethoscope, weighing machine, regular supply of IFA tablets, and vitamin A capsules, etc.

Two key issues highlighted by medical officers as needed to uplift the health situation of marginalized women were adequate nutrition and education (schooling). A perceived change was noticed in the functioning of community health workers, including need-based planning, timely planning for services, and an inclusive approach to prioritize the health needs of marginalized women.

*The key issues of women from marginalized families included poverty, migration, lack of education, ignorance, social outcasts, poor transport facilities, more belief in quacks, or traditional healers than registered practitioners. Superstitions,* etc. (A medical officer during IDI)

## Discussion

This study evaluated a community-based intervention on improving awareness and utilization of maternal and child health services and access to livelihood and savings by women.

The peer-led intervention seemed to have increased awareness of the women about maternal and child health services. Other studies with similar intervention designs have been effective in improving the health awareness of women [30, 31]. The participatory behavior change communication with women’s peers has emerged as a systematic approach to promote maternal and child health. Peer-led interventions are supported by theoretical frameworks of mutual support and self-help, social cognitive and social learning, social support, social comparison and networking, and empowerment [32].

There was a perceived change in the utilization of most maternal and child health services by women except for the consumption of IFA tablets and uptake of contraceptives. Low consumption of IFA tablets among women, despite health workers visiting them, has been reported in other studies [30, 33]. Plausible explanations for this were forgetfulness (non-compliance), perceived side effects of tablets among women, and low stocks of the tablets at health facilities [34, 35]. Nyugen et al., in their study in Bangladesh, reported significant effects on the receipt of free IFA and calcium supplements by pregnant women. This cluster randomized control trial had a multi-pronged approach that included nutrition-focused interpersonal counselling, community mobilization, and the distribution of free IFA and calcium supplements to women. According to the study, closed supervision by highly-trained health workers and performance-based incentives to heath volunteers contributed to the change in IFA consumption among women [36].

Contraceptive decision-making, to a great extent, is influenced by women’s financial autonomy and social status, socio-economic status of the family, and cultural and religious beliefs. Furthermore, early marriage, fear of side effects, partner’s low education, and lack of adequate supply and friendly attitude of health workers contribute to the low uptake of family planning services by women [37]. Our intervention has not addressed all these determinants at par. Further evidence-based interventions are warranted that target these determinants holistically and focus on men and women to increase the uptake of family planning services. A systematic review of 63 studies, ‘what works in family planning intervention,’ revealed significant improvements in knowledge, attitudes, discussion, and intentions by demand-side or supply-side programs; however, the impact on contraceptive use and use of family planning services was limited [38].

There has been a perceived change in the attitude of family members, including husbands and mothers-in-law, towards maternal health, which influenced the utilization rate of health services among women. A recently published systematic review assessing the effectiveness of community-based intervention concluded that the support of influential family members like husbands and mothers-in-law is crucial for bringing change in the health practices of women [39]. The quantitative analysis reported an increase in the percentage of women receiving postnatal care services, which contradicted to what was told by midwives in the interviews. This can be explained by the fact that we measured postnatal care uptake within 48 h of delivery, whereas midwives’ view might be based on the completion of seven postnatal care visits with every woman. Adherence to the postnatal care regimen is poor among women and has not been achieved in many similar interventions. Studies highlighted the need to engage men, educate women, and deliver home-based care to enhance the postnatal care [40, 41].

The uptake of jobs in MGNREGA or through other means increased post-intervention among women. Prior research indicated that MGNREGA has been successful in improving women’s empowerment, social and financial inclusion, especially of the vulnerable groups [42]. However, there were several challenges with the implementation of MGNREGA and its access by vulnerable populations. Findings from other studies reported that in Uttar Pradesh, despite increased participation of marginalized communities (scheduled castes or tribes, 48%), the participation by women in MGNREGA was meager (20%), which calls for policies and interventions to address the issues of empowerment and skill-building for increasing women’s participation [43, 44]. MGNREGA can be envisaged as a women’s economic and social empowerment program [45].

Women seemed to have learned to save money and use it for emergencies or their health purposes, such as transportation to the hospital at the time of delivery. However, less than 50% of women had opened an account on an average from the two districts. This might be due to poor understanding of banking or savings among women or a lack of support from the husband or family to do so, or limited availability or accessibility to banks in the rural areas. Neither the data regarding reasons for not having an account were obtained in the survey, nor were they asked during the interviews. This creates a gap in our understanding of the factors responsible for poor access to savings among women, which could be explored in future studies. A cross-sectional study from Rajasthan found that financial literacy, including behavior and attitude (savings, household budgeting, opening an account) of the women, especially from rural areas, was poor. One of the major factors attributable to this poor financial literacy could be their reliance on other family members, especially on males, for finance-related matters, which could be another reason for their non-involvement in investment decisions [46]. Financial literacy programs for women can be viewed as an effective approach to addressing education and financial needs.

A recent national-level survey reported that more than one-third of women from Uttar Pradesh had savings in the past 12 months, which have increased in the last 10 years [47]. Although there are gender disparities in the health care expenditures for care in India, the expenditure on women’s health has increased over the years, most of which was met from incomes/savings [48]. This underpins the importance of integrated health and financial literacy, besides improving access to employment for women. The evidence for a successful and convergent model of an intervention that aimed to address poverty and women’s empowerment in social spheres comes from a program implemented in the south of India, known as the *Kudumbashree* program. The program not only supported women for participation in income-generation activities but also helped them in gaining confidence, strength, and decision-making ability for their health [49].

The intervention had been perceived successful in transforming the functioning of VHSNC and seemed to have made the members of committees more responsible and knowledgeable about their responsibilities. Previous studies have highlighted gaps in the effective functioning of VHSNC and the need for building the capacity of their officials and improving their maturity [28, 50, 51]. Village health committees effectively decentralize health planning and action; however, they suffer from weak community linkages and participation because of a lack of inclusive membership, poor community engagement, and weak support from the health authorities [52]. This calls for the need to assist VHSNCs in planning effectively, allocating their funds rationally between health, nutrition, and sanitation activities in the community.

Some of the additional benefits of the intervention included a perceived change in gender equity norms in the communities wherein men realized the importance of a girl child and the harmful consequences of female foeticide. Women got a space in familial discussions and respect from their husbands. A recently published systematic review of 61 evaluations highlighted the need for multi-sectoral actions, multi-stakeholder involvement, diversified programming, and fostering social participation and empowerment to transform gender relations. This would promote equality and achieve health and wellbeing among people. The review acknowledged the importance of engaging the community and building social support systems besides generating awareness around restrictive gender norms in gender-transformative approaches [53].

While comparing some of the indicators between the two districts, it was realized that the improvement in Kaushambi was lesser than in Banda. Some of the factors attributed to lesser performance could be higher poverty, illiteracy, and unemployment in Kaushambi than Banda (as highlighted in the baseline study).

### Limitations and strengths

Although the findings bring to light the effects of the peer-led intervention, the results may be interpreted in view of certain limitations. The ‘post-test analysis of the project group’ research design cannot ascertain the efficacy of the intervention, and in the absence of a control group, its generalization and validity are limited. The project was limited to two districts of Uttar Pradesh, which were different from many other geographies. Both districts have a large population of marginalized people and limited availability of health services. The cultural and religious beliefs are deeply rooted, and extreme poverty is common to both districts. The end line evaluation was done using qualitative methodology, thereby limiting the quantifiable measurement of change in the health and economic status of women. The achievements of the project cannot be attributed solely to our intervention due to the concurrent actions of the government and other community-based organizations. Furthermore, the methods of investigations were different during the baseline or end line, and hence, it was not possible to compare their findings.

The community-based implementation across a large sample of women (*n* = 37,324) was its strength and was better than many randomized trials not reflective of true field settings. Randomized trials are often limited by a lack of external validity, explicit mechanisms of change, and feasibility, and acceptability of the experimental design [54]. Most of the field staff were women and from the same field settings, which helped in smoothly executing the intervention. The use of standardized tools leveraged easy adaptability and usage in the community.

## Conclusions

The study accomplished the objectives of the end line evaluation by exemplifying the perceived enhancement in the awareness and utilization of antenatal, postnatal, and child care services by women in the intervention sites. Moreover, increased access to livelihood, savings, and support of the stakeholders to women in accessing maternal health services was perceived in the evaluation. We perceived the fulfillment of the other two objectives, including improved functioning of the community institutions and the adoption of an inclusive approach by community health workers in the end line evaluation.

The intervention demonstrates that a peer-led approach may be used to improve the combined health and economic outcomes of marginalized women. Given the large number of social and structural determinants that affect health, targeting health factors alone may not be effective. Access to livelihood and savings are critical to women’s empowerment and the utilization of health services. Furthermore, the simultaneous engagement of husbands, mothers-in-law, health workers, and village health committees in the intervention is a promising sustainable strategy for health improvement in women. India’s flagship scheme, MGNREGA, is a novel step to ensuring livelihood security to the marginalized population, the effective reach, and successful implementation of which is still underachieved. The results provide optimism for improvement in the outcomes that did not change in the intervention, such as the IFA consumption, family planning uptake, and access to banking, provided actions are done with more rigor and intensity.

## Supplementary Information

**Additional file 1:** **Supplementary Figure 1.** The cover page and the table of contents’ page in the flipbook for beneficiaries. The table of contents displays the name of the 7 topics covered in the flipbook. The flipbook is in Hindi (local language). The instructions of how to use the book are shown behind the covers page. The image depicted above was developed by us. **Supplementary Figure 2.** The cover page and the table of contents’ page in the module. The table of contents displays the name of the 10 sessions in the module. The module is in Hindi (local language). The image depicted above was developed by us. **Supplementary Figure 3.** Progress tracking chart of the intervention activities across three intervals of the project (first 15 months, middle 12 months and last 8 months).

**Additional file 2:** **Supplementary Table 1.** District-wise distribution of the socio-demographic characteristics of the women (*n* = 476) at the rapid assessment survey. **Supplementary Table 2.** District-wise distribution of the prevalence of awareness and utilization of the maternal and child health services, and access to livelihood and savings by women (*n* = 476) at rapid assessment survey.

**Additional file 3.** Interview Guide for different Focus Group Discussions.

## Data Availability

The datasets used and/or analyzed in the present study are available from the corresponding author on reasonable request.

## Abbreviations

MMR: Maternal Mortality Ratio
MGNREGA: Mahatma Gandhi National Rural Employment Guarantee Act
SDG: Sustainable Development Goal
VHSNC: Village health, sanitation, and nutrition committee
