# Supplementary figures and images for: Evaluation of a community-based intervention for health and economic empowerment of marginalized women in India
Source: BMC Public Health. 2020 Nov 23;20:1766. doi: 10.1186/s12889-020-09884-y (PMC7686717; doi:10.1186/s12889-020-09884-y)

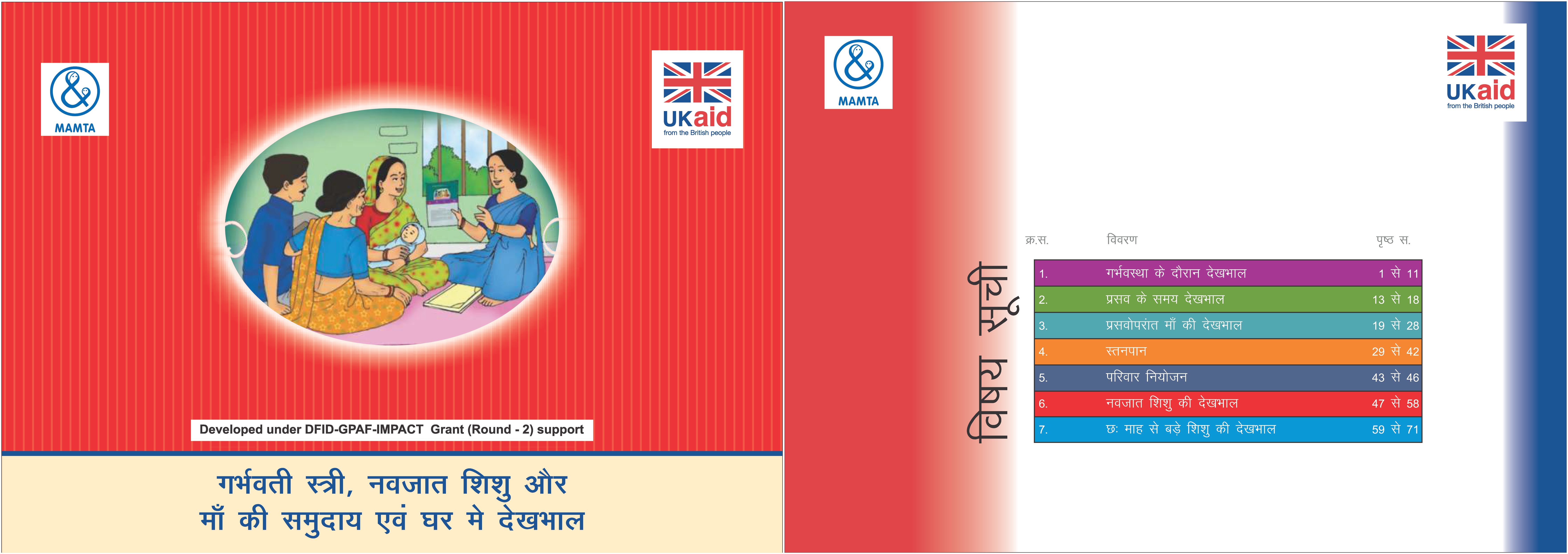

Supplement: Supplementary file 1 — Additional file 1: Supplementary Figure 1. The cover page and the table of contents’ page in the flipbook for beneficiaries. The table of contents displays the name of the 7 topics covered in the flipbook. The flipbook is in Hindi (local language). The instructions of how to use the book are shown behind the covers page. The image depicted above was developed by us. Supplementary Figure 2. The cover page and the table of contents’ page in the module. The table of contents displays the name of the 10 sessions in the module. The module is in Hindi (local language). The image depicted above was developed by us. Supplementary Figure 3. Progress tracking chart of the intervention activities across three intervals of the project (first 15 months, middle 12 months and last 8 months). [file 12889_2020_9884_MOESM1_ESM.zip › Supplementary Figure 1R5.jpg]

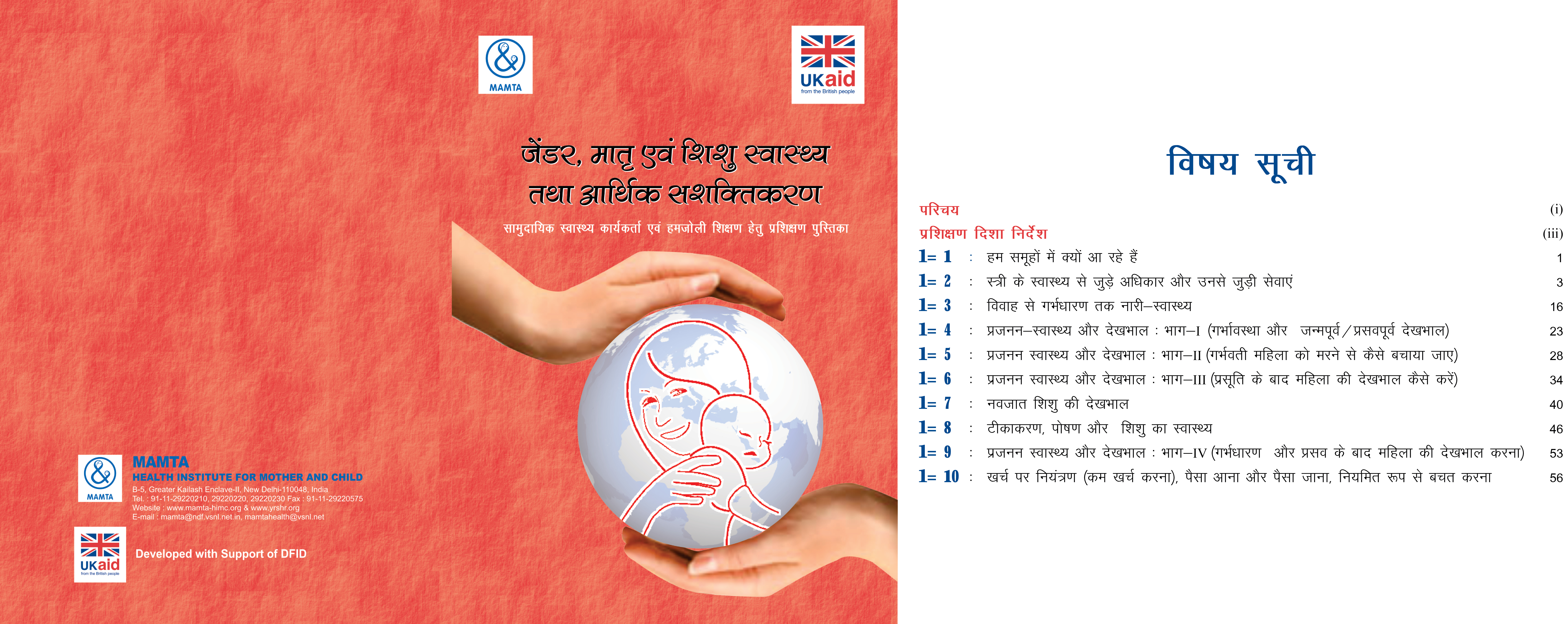

Supplement: Supplementary file 1 — Additional file 1: Supplementary Figure 1. The cover page and the table of contents’ page in the flipbook for beneficiaries. The table of contents displays the name of the 7 topics covered in the flipbook. The flipbook is in Hindi (local language). The instructions of how to use the book are shown behind the covers page. The image depicted above was developed by us. Supplementary Figure 2. The cover page and the table of contents’ page in the module. The table of contents displays the name of the 10 sessions in the module. The module is in Hindi (local language). The image depicted above was developed by us. Supplementary Figure 3. Progress tracking chart of the intervention activities across three intervals of the project (first 15 months, middle 12 months and last 8 months). [file 12889_2020_9884_MOESM1_ESM.zip › Supplementary Figure 2R5.jpg]
