# Supplementary material for: Evaluation of a community-based intervention for health and economic empowerment of marginalized women in India
Source: BMC Public Health. 2020 Nov 23;20:1766. doi: 10.1186/s12889-020-09884-y (PMC7686717; doi:10.1186/s12889-020-09884-y)
Supplement: Supplementary file 2 — Additional file 2: Supplementary Table 1. District-wise distribution of the socio-demographic characteristics of the women (n = 476) at the rapid assessment survey. Supplementary Table 2. District-wise distribution of the prevalence of awareness and utilization of the maternal and child health services, and access to livelihood and savings by women (n = 476) at rapid assessment survey. [file 12889_2020_9884_MOESM2_ESM.docx]

**Supplementary Table 1.** **District-wise distribution of the socio-demographic characteristics of the women (n=476) at the rapid assessment survey**

| **Socio-demographic variables** | **Banda (n=240)**  **N(%)** | **Kaushambi (n= 236) N(%)** |
| --- | --- | --- |
| Age groups (years) | | |
| 15-24 | 82 (34.2) | 56 (23.8) |
| 25-35 | 140 (58.3) | 165 (69.8) |
| >35 | 18 (7.5) | 15 (6.4) |
| Mean age of the women (years) | 27.6 | 27.6 |
| Mean age at marriage (years) | 17.2 | 17.3 |
| Mean number of children per women | 3 | 3 |
| Religion | | |
| Hindu | 220 (91.3) | 230 (97.5) |
| Muslim | 20 (8.8) | 6 (2.5) |
| Social class |  |  |
| Scheduled castes | 196 (81.7) | 204 (86.4) |
| Scheduled tribes | 9 (3.8) | 12 (5.1) |
| Other marginalized castes | 33 (13.8) | 19 (8.1) |
| Non-marginalized class | 2 (0.8) | 1 (0.4) |
| Education status of the women | | |
| Illiterate | 157 (65.8) | 191 (80.9) |
| Literate but never been to school | 9 (4.2) | 7 (2.5) |
| Literate (formal education) | 74 (30.8) | 38 (16.5) |
| Current employment status of the women | | |
| Work in their own field | 7 (2.9) | 24 (10.2) |
| Work as wager in other’s field | 63 (26.1) | 180 (76.6) |
| Employed under MGNREGA | 11 (4.2) | 0 |
| Housewife | 132 (55.0) | 27 (11.1) |
| Others | 27 (10.9) | 5 (2.1) |

*Abbreviations: MGNREGA: Mahatma Gandhi National Rural Employment Guarantee Act*

**Supplementary Table 2. District-wise distribution of the prevalence of awareness and utilization of the maternal and child health services, and access to livelihood and savings by women (n=476) at rapid assessment survey**

| **Variables** | **Banda (n=240)**  **N(%)** | **Kaushambi (n=236)**  **N(%)** |
| --- | --- | --- |
| **Awareness of maternal and child health care** | **Yes** | **Yes** |
| Women who heard about early registration of pregnancy | 179(74.6) | 138(58.6) |
| Women who heard about anemia during pregnancy | 49(20.4) | 34(14.4) |
| Women who were aware of the maternity benefit scheme | 85(35.4) | 133(56.4) |
| Women who heard about postnatal care services | 73(30.4) | 48(20.3) |
| Women who heard about family planning methods | 46(19.2) | 32(13.6) |
| Awareness on home-based management of diarrhea  Give oral rehydration solution  Salt and water  Normal food to continue  Continue breastfeeding  Sufficient fluids | 66(27.5)  60(25.0)  9(3.8)  24(10.0)  30(12.5) | 42(17.8)  105(44.5)  3(1.3)  8(3.4)  4(1.7) |
| Awareness about symptoms of pneumonia  Difficulty in breathing  Difficulty in eating  Excessive yawning and difficulty in getting up  Chest pain or cold  Fast breathing  Running nose | 202(84.5)  77(32.1)  79(33.3)  160(66.7)  97(40.5)  108(45.2) | 158(67.1)  18(7.6)  33(13.9)  63(26.6)  108(45.6)  3(1.3) |
| Heard about child immunization | 196(81.7) | 161(68.2) |
| Women who know about safe places for abortion | 58(24.2) | 70(29.7) |
| Women who heard about the national health insurance scheme | 83(34.6) | 56(23.6) |
| Women who heard about the MGNREGA scheme | 101(42.1) | 109(46.1) |
| **Utilization of maternal and child health services** |  |  |
| Women who received 3 and/or more antenatal check-ups | 60(25.0) | 46(19.7) |
| Women who received 2 tetanus toxoid injections during pregnancy | 228(95.0) | 199(84.3) |
| Women who received 100 iron-folic acid tablets | 187(77.9) | 158(66.9) |
| Women who had a urine test done during pregnancy | 164(68.3) | 43(18.2) |
| Women who had any blood test done during pregnancy | 184(76.7) | 75(31.8) |
| Women who saved money for the delivery | 86(35.8) | 122(51.7) |
| Women who had an institutional delivery | 190(79.2) | 164(69.6) |
| Women who received money from the maternity benefit scheme | 203(84.7) | 171(72.7) |
| Women who received two or more postnatal services from the health facility | 108(45.1) | 41(17.6) |
| Women who initiated breastfeeding within an hour of birth | 219(91.3) | 101(43.0) |
| Children who were fully immunized | 98(40.8) | 87(36.9) |
| Women who were currently using any family planning method | 55(23.0) | 31(13.0) |
| Women who ever had any abortion | 13(5.4) | 10(4.3) |
| **Others** |  |  |
| Women who possessed a bank account | 93(38.8) | 108(45.8) |
| Women who possessed the national health insurance card | 36(15.0) | 14(6.0) |
| Women who were registered to work under the MGNREGA scheme | 73(30.4) | 67(28.4) |

*Abbreviation: MGNREGA: Mahatma Gandhi National Rural Employment Guarantee Act*

*The percentages may not add up to a total of 100% because the frequencies of only ‘yes’ responses have been shown.*
