# Supplementary material for: Evaluation of a community-based intervention for health and economic empowerment of marginalized women in India
Source: BMC Public Health. 2020 Nov 23;20:1766. doi: 10.1186/s12889-020-09884-y (PMC7686717; doi:10.1186/s12889-020-09884-y)
Supplement: Supplementary file 3 — Additional file 3. Interview Guide for different Focus Group Discussions. [file 12889_2020_9884_MOESM3_ESM.docx]

**Interview Guide for different Focus Group Discussions**

1. **FGD Tool for community health workers**

Q1: (a) What health problems do women of reproductive age group suffer from?

(b) Do you think that women of Scheduled Castes/Scheduled Tribes’ families face this problem more than the women of other groups?

(C) Do you pay more attention to resolve the health issues of marginalised group?

Q2: (a) What health services are you providing to the women of reproductive age group?

(b) Do you feel that all the medical facilities are provided to the women of marginalised group?

Q3: (a) Do you feel that mothers are utilising immunization and other medical facilities in proper way? Are they deprived of these facilities on the basis of their caste or religion?

(b) What special efforts have been done to reach to backward classes?

Q4: Do you find any lacunae in providing medical services to Backward (marginalised) groups? Do you feel that there is a need to improve upon it? If yes, how?

Q5: Do you feel that less number of women are utilising health services. Approximately, what percentage of women are availing MCH services and what could be the cause of low utilisation? Do the women who are not availing health services belong to a particular caste or religion?

Q6: (a) Do the pregnant women follow the advice given to them about nutrition? If yes, how do you ensure it? If not, why don’t they follow the advice?

(b) How do you motivate women of marginalised group to take good nutrition?

Q7: Do you find any change in utilisation of MCH services in last one year? If yes, what could be the reason of it?

Q8: How do you share information with women regarding child care? Do you give special attention to the women of marginalised group in this regard? What problems do you face while sharing the information with them and what strategy do you follow to minimise these issues?

Q9: Has MAMTA NGO did any work in your region (area)? What is your opinion about it? Do you want to give any suggestion to improve upon their functioning?

Q10: (a) How do you impart knowledge of life skills and gender equity to the families of marginalised groups?

(b) How do you provide them benefits through VHSNC?

Q11: How are emergency services like arranging blood, vehicle or money dealt with?

1. **FGD Tool for BENEFICIARIES**

Q1: Do you work to increase your family income? If yes, how many days do you work in a year?

Q2: How many of Below the Poverty Line families have bank account? What kind of help do you get from the project? Do you think that bank account is essential for mother and child health?

Q3: What do you understand by savings? How do you ensure small scale daily savings in your family?

Q4: Have you heard about *Janani Suraksha Yojana*? How many women have been benefitted by this in the recent years?

Q5: Did you get the information about life skills and gender discrimination through health employees?

Q6: Are you a member of any women group? If yes, do you find yourself strong after joining the group?

Q7: What benefits do you get after joining the women group?

Q8: How are the group sessions on MCH and financial strengthening helpful to you?

Q9: Do you share the information that you get from these sessions, with your family members?

1. **FGD Tool for MIL (mother-in-Law)**

Q1: Have the groups of women been formed under any project in your village in which women of your home are also included? If yes, in your opinion, how are they benefited?

Q2: Whether, any special attention is given to particular class while making groups? If yes, what are those classes?

Q3: Do you think that there is a change in utilisation of MCH services in last few years? If yes, explain.

Q4: Which services are being provided to the women of reproductive age group? Whether any special services are being provided to the women of backward classes? If yes, explain them.

Q5: Whether, any special information about life skills and gender discrimination are being shared with the women of the special group? If yes, what are these information?

Q6: Do you feel that special attention is given to resolve reproductive/nutrition or any other health related problems of these special groups? If yes, how are these issues resolved?

Q7: What role do you play to make the health services available to the women of your family?

Q8: Has some special efforts been done under the project to do the above mentioned work successfully?

Q9: Do you appreciate the work done under the project? What is your opinion about further running and developing this work of project?

1. **FGD Tools for PEER EDUCATORS (PEs)**

Q1(a) What is your role in this project as a peer educator?

(b) How important do you consider your role and why?

Q2: What problems do you face while playing this role and what do you do resolve it?

Q3: Do you think that the training given to you to play your role, is adequate? If yes, how?

Q4: Do you feel that you need more training?

Q5: How these training sessions be made more beneficial/effective?

Q6: In your opinion, which sessions were more difficult to understand or to deal with?

Q7: What positive changes could you bring about in your groups?

Q8: (a) Have you ever tried to find out how many women of your group have been benefited by the information shared with them and how many of them have been facing problems?

(b) What steps have been taken by you to resolve these problems?

Q9: How do you ensure that group is being benefited by government services?

Q10: What changes do you observe in the community after the intervention of project?

Q11: How do you ensure that the problems of pregnant women of your group have been resolved properly?

Q12: How do you put up the issues of your group to the concerned official?

1. **FGD of PROJECT STAFF (outreach workers)**

Q1: How long you have been associated with the project?

Q2: In your opinion, what are the main objectives of the project?

Q3: (a) What procedure do you follow for making groups of women?

(b) How many groups of women are there in your region?

Q4: What special efforts have been done by you for the welfare of women of marginalised groups?

Q5: What special plans are there for the women of reproductive age group?

Q6: On which specific areas have you given the training to health workers? How much beneficial is it for them?

Q7: What steps have been taken by you to improve MCH services further?

Q8: What steps have been taken to make women socially and economically self-reliant?

Q9: What efforts have been done to develop life skills and to develop awareness about gender equity? Is any special attention given to a particular class?

Q10: Do government health officials extend their help? If yes, how do they help?

Q11: Do MGNREGA officials cooperate with you? If yes, how?

Q12: Being a part of the project, how much successful do you find yourself and also the project? What is your suggestion to carry on this project ahead?

**Interview guides for different In-depth Interviews (IDI)**

1. **IDI Tool for PEER EDUCATOR**

Q1: (a) What is your role in the project as a peer educator?

(b) Do you consider your role very important? Why?

Q2: What is your role in the project to improve social and financial conditions of women of marginalised section?

Q3: Do you find any improvement in social and economic conditions of women of marginalised sections? If yes, what changes do you observe?

Q4: Did you ever try to give information or services to the women of reproductive age group? If yes, how?

Q5: Do you feel that special efforts have been taken to meet the reproductive/nutritional or health needs of women of marginalised group?

Q6: Is there any change in the pattern of availing MCH services in last two years? If Yes, what kind of changes do you observe and what is your role in it?

Q7: How did you help in sharing formation with group or in giving training to the groups?

Q8: What problems do you face in running the sessions or playing your role? How do you resolve such issues?

Q9: Is the training given to you to play your role, is adequate? If yes, how?

Q10: Do you feel that you need more training? If yes, on what issues?

Q11: In your opinion how these training sessions be made more effective?

Q12: In your opinion, which training sessions were more difficult to comprehend and to deal with and why?

Q13: What positive changes could you bring about in your group?

Q14: (a) Have you ever tried to find out how many women of your group have been benefited by the information shared with them and how many of them have been facing problems?

(B) What steps have been taken by you to resolve these problems?

1. **IDI Tool for midwives**

Q1: How do you identify socially and economically backward women? Please explain the criteria.

Q2: What problem do you face in identifying them?

Q3: How do you motivate women of socially and economically backward class to utilise health services?

Q4: How do you make groups of socially and economically backward class?

Q5: How many groups of such women did you make in last one year?

Q6: When did you get the training to work on the people of socially and economically backward class?

Q7: I would like to know about the quality of training sessions given by project staff. Rate the training sessions on the scale of 1 to 10 where 1 represents the worst and 10 represents the best quality.

Q8: (a) Do you feel that in your region regarding utilisation of services, the ratio of women of backward class families is less than that of general class?

(b) Have you ever noticed that people of backward classes face the difficulties in access of health services? What difficulties do they face and why?

Q9: (a) Mention two main health issues of women of underprivileged group of your region. How do you identify their needs?

(b) Have you developed any technique/plan to work on it?

Q10: Do the women of backward classes have the knowledge of their right to health? Do they practice it to avail health facilities?

Q11: (a) In your opinion, how much knowledge of ANC do pregnant women have?

(b) Do you find any change in the awareness in women of weaker section?

Q12: (a) What percentage of women of backward classes go for institutional delivery?

(b) State the factors that are creating obstacles in providing services to them.

(c) Do you think that this situation can be improved? If yes, how?

Q13: (a) What steps have been taken to provide better PNC services to the women of backward classes?

(b) Do you ensure that the women of backward classes should go under all kind of health check-ups after the delivery?

(c) How can they be encouraged to go for PNC check-ups?

Q14: How do you ensure in routine that the benefits/services are reaching to the women of socially and economically backward classes?

Q15: (a) Do you find any scope of improvement in the process of providing services to the women of backward classes?

(b) If yes, what steps need to be taken to improve upon it?

Q16: (a) What changes you have been experiencing after the association with the project?

(b) What else can be done for the betterment of women of backward classes and how?

Q17: Are the VHSNC meetings conducted regularly? Have the issues related to the women of backward classes been discussed? If yes, explain.

Q18: Have you ever been called by MGNREGA officials to attend the meeting and discuss the issues related to people of backward classes?

Q19: In your region, who is the decision maker in family?

Q20: What is the “Gullak Culture”? What do you do in this regard? Express your views?

Q21: Do you find intervention beneficial? Justify your answer.

1. **IDI Tool for community health workers**

Q1: How do you identify socially and economically backward women? Please explain the criteria.

Q2: What problem do you face in identifying them?

Q3: How do you motivate women of socially and economically backward class to utilise health services?

Q4: How do you make groups of socially and economically backward class?

Q5: How many groups of such women did you make in last one year?

Q6: When did you get the training to work on the people of socially and economically backward class?

Q7: I would like to know about the quality of training sessions given by project staff. Rate the training sessions on the scale of 1 to 10 where 1 represents the worst and 10 represents the best quality.

Q8: (a) Do you feel that in your region regarding utilisation of services, the ratio of women of backward families is less than that of General class?

(b) Have you ever noticed that people of backward classes face the difficulties in access of health services? What difficulties do they face and why?

Q9: (a) Mention two main health issues of women of underprivileged group of your region. How do you identify their needs?

(b) Have you developed any technique/plan to work on it?

Q10: Do the women of backward classes have the knowledge of their right to health? Do they practice it to avail health facilities?

Q11: (a) In your opinion, how much knowledge of ANC do pregnant women have?

(b) Do you find any change in the awareness in women of weaker section?

Q12: (a) What percentage of women of backward classes go for institutional delivery?

(b) State the factors that are creating obstacles in providing services to them.

(c) Do you think that this situation can be improved? If yes, how?

Q13: (a) What steps have been taken to provide better PNC services to the women of backward classes?

(b) Do you ensure that the women of backward classes should go under all kind of health check-ups after the delivery?

(c) How can they be encouraged to go for PNC check-ups?

Q14: How do you ensure in routine that the benefits/services are reaching to the women of backward classes?

Q15: (a) Do you find any scope of improvement in the process of providing services to the women of backward classes?

(b) If yes, what steps need to be taken to improve upon it?

Q16: (a) What changes you have been experiencing after the association with the project?

(b) What else can be done for the betterment of women of backward classes and how?

Q17: Are the VHSNC meetings conducted regularly? Have the issues related to the women of backward classes been discussed? If yes, explain.

Q18: Have you ever been called by MGNREGA officials to attend the meeting and discuss the issues related to people of backward classes?

Q19: In your region, who is the decision maker in family?

Q20: What is the “Gullak Culture”? What do you do in this regard? Express your views?

Q21: Do you find intervention beneficial? Justify your answer.

1. **IDI Tool for HUSBAND**

Q1: Do you know that some project is running in your village? If yes, when did it commence?

Q2: Has anyone from this project ever contacted you, your wife or any other family member? If yes, how many times did they contact you?

Q3: Which subjects do the project employees discuss with you? Explain in detail.

Q4: Are some special programmes for the women of backward classes being run?

Q5: (a) Is your wife a member of any group made under the project? If yes, how is she benefited by it?

(b) Do you have any problem because of this that your wife is member of the group?

Q6: What cooperation is being given by the project employees to make the MCH services available to the women of your village?

Q7: Do you get the cooperation from the project employees if your wife or any other family member suffers from any health problem? If yes, how do they help?

Q8: In your opinion, is any special information about life skills or gender discrimination being shared under this project? If yes, give the details of it.

Q9: How can you support your family through this project?

Q10: Do you find any lacunae in the functioning of the project? If yes, explain it.

Q11: What is your suggestion to run the project successfully?

1. **IDI Tool for MEDICAL OFFICER**

Q1: Do you find that in availing government health benefits, the number of women of marginalised category is less than that of general category?

Q2: Why is it so?

Q3: What are the common health issues of the women of backward classes of your region?

Q4: Why are these issues important and can they be resolved?

Q5(a) Do you know that groups of women of marginalised section have been made under the project?? What difficulties did they come across while making group?

(b) How did they make groups? Did you help them in making of groups?

Q6: How effective were the training sessions of project staff on MCH and financial literacy?

Q7: Could the target group understand that socio-economic independence is essential for better MCH care? Did they start saving money?

Q8: Did you ever notice in your hospital /PHC that the women of marginalised families were also given a chance to participate in decision making activity in family matter?

Q9: Have the important /responsible persons like PRIs/ VHSNC members been included in health programmes?

Q10: Do you feel that the project has created the environment for Health dept and MGNREGA to work together? Whether the two departments now able to coordinate with each other?

Q11: Do the women of marginalised group have all the information about ANC/PNC/ hospital delivery etc.? Do they know all about health services?

Q12: Has the information about MCH care been explained to the family members of marginalised group to that extend that now they take proper care of pregnant woman and readily cooperate with her?

Q13: (a) Do you find any change in the thinking of family members of pregnant woman of marginalised group? Do family members take due care of her irrespective of gender?

(b) What steps need to be taken to improve the status of women?

Q14: What else can health workers do for ANC/PNC care specially of women of marginalised group?

Q15: How do you ensure in routine that the health services are reaching to the marginalised group?

Q16: What is your suggestion regarding increase participation of men in MCH care?

Q17: What important lessons did you learn from the project?

1. **IDI Tool for VHSNC members**

Q1: Do you know that a project is running/functioning in your village? From which date is it operational?

Q2: Has any special consideration been given by the project for the families of particular categories? If yes, share some information about it.

Q3: Do you know the criteria on the basis of which they have been given special consideration?

Q4 What steps have been taken so far by the project staff to provide MCH services to the women of reproductive age group?

Q5: How this project is supporting the women of marginalised section in resolving the issues related to their delivery, nutrition and health?

Q6: What steps have been taken by the project to develop opportunities for life skills in your village?

Q7: What steps have been taken by the project to develop awareness about gender equity?

Q8: What role do you play in the activities done by the project to improve upon the financial conditions of marginalised group?

Q9: Have some groups of women been formed by the project employees? If yes, what were the criteria of forming groups and how many such groups are there in your village?

Q10: Has the project been putting some effort to improve the functioning of VHSNC & health day celebration in your village?

Q12: After the intervention, do you find any increase in number of women of marginalised group availing MCH services?

Q13: Do you find any lacunae in the functioning of the project?

Q14: Do you want to give any suggestion to the project employees to make the intervention more effective?

1. **IDI Tool for PROJECT STAFF (outreach workers)**

Q1: How long you have been associated with the project?

Q2: In your opinion, what are the main objectives of the project?

Q3: (a) What procedure do you follow for making groups of women?

(b) How many groups of women are there in your region?

Q4: What special efforts have been done by you for the welfare women of marginalised group?

Q5: What special plans are there for the women of reproductive age group?

Q6: On which specific areas have you give the training to health workers? How much beneficial is it for them?

Q7: What steps have been taken by you to improve MCH services further? How do you ensure the availability of services to the beneficiaries?

Q8: What steps have been taken to make women socially and economically self-reliant?

Q9: What efforts have been done to develop life skills and to develop awareness about gender equity? Is any special attention given to a particular class?

Q10: Do government health officials extend their help? If yes, how do they help?

Q11: Do MGNREGA officials cooperate with you? If yes, how?

Q12: Being a part of the project, how much successful do you find yourself and also the project? What is your suggestion to carry on this project ahead?
